# RDX and miRNA Expression: Zhang and Pan Respond

**DOI:** 10.1289/ehp.0800276R

**Published:** 2009-03

**Authors:** Baohong Zhang, Xiaoping Pan

**Affiliations:** Department of Biology, East Carolina University, Greenville, North Carolina, E-mail: zhangb@ecu.edu; Department of Chemistry, Western Illinois University, Macomb, Illinois

We appreciate the interests and comments from Bannon et al. regarding our recent article on RDX-induced aberrant microRNA (miRNA) expression in mice (Zhang and Pan 2009). However, we disagree with their comments based on the misunderstanding of our results and conclusions.

The objective of our study (Zhang and Pan 2009) was not to use clinical chemistry and histopathological features to diagnose neoplasm and carcinogenesis, but to investigate the effect of a low-dose RDX exposure on miRNA expression in mice. Our results show that exposure to RDX at 5 mg/kg in the diet for 28 days induced aberrant expression of miRNAs in B6C3F1 mice. Our discussion of the implications of altered miRNAs in the carcinogenic risk of RDX suggested by a previous study (Lish et al. 1984) are based on the knowledge that aberrant miRNA expression is associated with a broad range of cancers (Zhang et al. 2007). Meanwhile, we also discussed the potential anticancer effects of RDX in our article. Results in our Figure 6 indicate that RDX exposure induced miR-206 expression, which may inhibit expression of *TNKS* (tankyrase, TRF1-interacting ankyrin-related ADP-ribose polymerase). The inhibition of TNKS causes telomere shortening and apoptosis, which inhibits carcinogenesis, and has thus been proposed as a potential cancer therapy (Seimiya 2006). Thereby, the miR-206 overexpression induced by RDX may provide a mechanism to prevent carcinogenesis.

Animals are more sensitive to chemical exposure at the gene level than at the physiologic level, and gene expression profile is a more powerful predictor of the outcome of disease than standard systems based on clinical and histologic criteria (van de Vijver et al. 2002). However, aberrant gene expression may or may not cause carcinogenesis. Cancer patho genesis is a complex process associated with aberrant expression of many genes. Recently identified miRNAs play critical roles in cancer development; overexpression or down-regulation of a single miRNA could influence cancer cell growth, invasion, and metastasis (Ma et al. 2007; Takamizawa et al. 2004). Also, more and more evidence demonstrates that environmental carcinogens cause aberrant expression of a suite of miRNAs. For example, Kalscheuer et al. (2008) recently demonstrated the differential expression of miRNAs in early-stage neoplastic transformation in the lungs of F344 rats chronically treated with the tobacco carcinogen NNK (4-(methylnitrosamino)-1-(3-pyridyl)-1-butanone). Thus, carcinogen-induced aberrant expression of miRNAs may be associated with carcinogenesis.

Brain-derived neurotrophic factor (BDNF) is targeted by multiple miRNAs. Besides miR-206, two experimentally validated BDNF-targeting miRNAs, miR-30a and miR-195 (Mellios et al. 2008), were also significantly up-regulated in mouse brain after RDX exposure. Thus, it is reasonable to suggest that miRNA-mediated BDNF expression is a neurotoxic effect of low RDX exposure, given that BDNF is an important neuro trophin supporting neuronal survival and differentiation, neurite outgrowth, and synaptic plasticity.

Seizures have been induced by RDX at high acute doses but not at low doses (Burdette et al. 1988; Schneider et al. 1978). Our result is similar to that in previous reports, and we observed no seizures in our study using low doses. Both proepileptogenic and antiepileptogenic effects of BDNF have been reported (Binder et al. 2001); therefore, we did not discuss the potential inhibition of BDNF in the context of RDX-induced seizure in our article. However, the up-regulation of BDNF reported in most chemical-induced seizures have been measured at postseizure and postlesion periods instead of pre seizure periods (Binder et al. 2001; Nawa et al. 1995). Indeed, BDNF is naturally down-expressed in chronic epileptic stages (Shetty et al. 2003), and the reduced level of BDNF may play important roles in the proexcitotoxic effect of chronic stress (Mattson 2007). Neurotoxicants such as alcohol (Fattori et al. 2008) and poly-brominated diphenyl ethers (Viberg et al. 2008) also inhibit BDNF expression. Alcohol has also been found to increase miR-335 expression at low doses but suppress miR-335 at high doses, suggesting some miRNAs respond to neurotoxicants in a dose-specific manner (Sathyan et al. 2007).

Our study (Zhang and Pan 2009) provides substantial information on miRNA expression as a phenotype in response to RDX exposure in mice. We reported many important novel findings that enlighten future research. For example, miR-206—belonging to the same family as miR-1, which regulates signal transduction at neuro muscular junctions (Simon et al. 2008)—was significantly up-expressed in our study. Therefore, elucidating the role of miR-206 in RDX-related neurotoxicity is an enormous project.
